# 

**Published:** 1917-09-29

**Authors:** 


### Passing Events and Topics.

### Gifts of Buildings.

The Bank House Estate, Congleton, Cheshire, comprising a mansion and 25 acres of ground, has been presented by Mrs. Little, the widow of Dr. David Little, of Congleton, for use as a home for disabled soldiers. At Preston, in the adjoining county of Lancashire, Mr. W. Birtwistle has presented a new hostel for the nurses and staff at the Moor Park V.A.D. Hospital.

### Walthamstow's Hospital Efforts.

Walthamstow and district appear to be setting a good example in the matter of not allowing the support of civil hospital work to diminish, in spite of war conditions, if we may judge by the recent reports of local efforts in this direction. The collection for the Hospital Saturday Fund in Walthamstow and district for the past year is now stated to be £422 12s. 8d., an increase of £16 13s. 4d. over the previous year. The fête recently organised and held at the county cricket ground, Leyton, in aid of the Walthamstow Hospital was an unqualified financial success, and eclipsed all former records, the first instalment only of the proceeds, a cheque for £505, exceeding any previous year's amount.

### Training in Dispensing.

Special day and evening courses, the former including bookkeeping and typewriting, in addition to pharmacy, chemistry, and materia medica, have been arranged at the South-Western Polytechnic Institute for women students desiring to pass the Assistants' Examination of the Society of Apothecaries, which qualifies them to act as dispensers to a medical practitioner and also, under certain conditions, in a hospital. Particulars of fees and hours may be obtained from the Secretary of the Institute (Room 78), Manresa Road, Chelsea.

### Matrons' and Sisters' Appointments.

Craghead Military Hospital, Bournemouth.—Mrs. Janet D. Dalgleish has been appointed matron. She was trained at the Dundee Royal Infirmary, and during the Boer War was matron of the Concentration Camp Hospital, Kimberley, South Africa, and later matron of the Kroonstad and District Hospital, South Africa. During the present war she has been temporary matron of the Halstead Cottage Hospital, Essex, and later matron of the 25th Durham V.A. Hospital, Ashburne, Sunderland.

Swanage, Cottage Hospital.—Miss Florence K. Samson has been appointed matron. She was trained at the General Hospital, Birmingham, and took a course in housekeeping at the Lord Mayor Treloar Hospital, Alton. She has been ward sister at Salisbury Infirmary and Birmingham General Hospital.

Manchester Babies' Hospital.—Miss Kate Cameron has been appointed lady superintendent. She was trained at Carlisle Infirmary, and has since been Queen's district nurse at Lochaber, and matron of the Victoria Red Cross Hospital, Stretford, Manchester.

Auxiliary Hospital, Helsby, Cheshire.—Miss M. Edwards has been appointed night sister. She was trained at the Denbighshire Infirmary, and has since been sister-in-charge at the Llandyrnog Red Cross Hospital, Denbigh.

East London Hospital for Children, Shadwell, E.—Miss Doris Markham has been appointed sister. She was trained at the East London Hospital for Children and at St. Bartholomew's Hospital, London.

East Preston Union Infirmary, Angmering.—Miss Sarah A. Lewis has been appointed ward sister. She was trained at Crumpsall Infirmary, Manchester, and has since been ward sister, superintendent nurse, and night sister at Plymouth, Wolverhampton, Barnsley, Congleton, Bishop's Stortford, and Brighton Union Infirmaries.

Forest Hospital, Buckhurst Hill, Essex.—Miss Butler has been appointed sister in charge. She was trained at St. George's Hospital, Hyde Park Corner, and has since been sister at the Florence Nightingale Hospital, Norwood Cottage Hospital, of a ward for wounded soldiers at Birmingham General Hospital, and assistant matron at Warneford General Hospital, Leamington Spa.

Hertford County Hospital.—Miss Mathews has been appointed sister of the massage and electrical department, and Miss Margaret Fairall night sister. Miss Mathews was trained at Walsall and District Hospital, and has since been staff nurse at the National Hospital for Epilepsy, Queen Square, Bloomsbury; Miss Fairall was trained at Salop Infirmary, Shrewsbury, and has since done private nursing.

**NOTE.—Particulars of Appointments for insertion in this column will be welcomed.**

### The Medical Schools.

Middlesex Hospital.—The opening of the medical school will take place on Monday, October 1, at 3 p.m., when the prizes will be distributed by Lady Cicely Gathorne Hardy. Owing to the war no formal invitations will be issued, but all who are interested in the hospital and its medical school are cordially invited to be present and to bring their friends. The annual dinner will not be held.

Charing Cross Hospital Medical School.—The distribution of prizes will be made in the out-patients' hall of the hospital (entrance at corner of King William Street) on Monday, October 1, at 3.30 p.m., by the Right Honourable Christopher Addison, M.D., M.P., Minister of Reconstruction. Tea and coffee will be served afterwards, and a reception will be held at the school, when the laboratories, museum, library, etc., will be open to inspection.

### Obituary.

The death has occurred, at the age of fifty-eight, of Mr. W. A. W. Price, secretary of the Herefordshire General Hospital since 1902.

Captain Henry C. Taylor, R.A.M.C., at one time senior house surgeon at Huddersfield Infirmary, has died on service at Netley Hospital, where he was in charge of a department.

### Gifts and Bequests.

Sir Charles Stamp Milburn, of Newcastle, whose death occurred in July, left £13,750 to charitable institutions, among the beneficiaries being the following:—Newcastle Infirmary, £3,000; Newcastle Hospital for Sick Children, £1,000; Newcastle Workshops for the Adult Blind, £1,000; Alnwick Infirmary, £500; and Dr. Barnardo's Homes (Washington Branch), £500.

The Manchester Royal Infirmary has received a legacy of £500 from the executors of the late Mary Barnes.

The women of Newfoundland have contributed £559 to the Edith Cavell Homes of Rest for Nurses.

Mr. and Mrs. S. G. Asher have given a complete *x*-ray apparatus and outfit to the Ascot Military Hospital, with the understanding that after the war it is to be transferred to the Royal Victoria Nursing Home at South Ascot.

For the purpose of endowing a cot in memory of the late Duke of Argyll, Princess Louise, Duchess of Argyll, has sent £500 to the Victoria Hospital for Children, Chelsea.

Mr. Thomas Shepheard, of Kingsley, Bournemouth, left £100 to Chester Royal Infirmary.

Mr. Walter Abbott Paynter, of Staines, left £2,000 towards a cottage hospital at Staines.

Subject to a life interest to his widow, Mr. John Smedley, of Belper Road, Derby, left £1,000 to the Derby Royal Infirmary (for the endowment of a cot to be known as the "John Smedley, J.P., Belper Cot"); £500 to the Derby Children's Hospital; and £300 to the Derby Deaf and Dumb Institute.

RATES OF SUBSCRIPTION: Twelve months, 6/6; Six months, 4/-; three months, 2/-, post free to any address in the United Kingdom. Abroad, Twelve months, 8/8; Six months, 5/-; Three months, 2/6, post free. THE HOSPITAL "Index" to Volume LXI., October 1916 to March 1917, is now ready, price 2½d. per copy, post free. Address The Manager, THE HOSPITAL, "The Hospital" Building, 28/29 Southampton Street, Strand, London, W.C. 2.

